# The Examination of the Sick; the Principles of Diagnosis; Therapeutic Methods, Etc.

**Published:** 1874-12-01

**Authors:** N. S. Davis


THE
### MEDICAL EXAMINER.
A
*Semi-Monthly Journal of Medical Sciences.*

EDITED BY N. S. DAVIS, M.D., AND F. H. DAVIS, M.D.

No. XXIII. CHICAGO, DEC. 1, 1874. VOL. XV.

### Original Communications.

### THE EXAMINATION OF THE SICK: BY INSPECTION, ORAL QUESTIONS, PALPATION OR TOUCH, INSTRUMENTAL AID; THE PRINCIPLES OF DIAGNOSIS; THERAPEUTIC METHODS, ETC.

A LECTURE IN THE REGULAR COURSE ON PRACTICAL MEDICINE, IN CHICAGO MEDICAL COLLEGE, BY N. S. DAVIS, M.D.

ONE of the most delicate and important duties of the physician is to examine his patient. The object of such examination is, to ascertain the location, extent, nature, stage of progress, and coincident derangements, of whatever disease or diseases may afflict the patient; together with the causes that may have been efficient either in producing it or perpetuating its existence. To accomplish this object fully, requires on the part of the practitioner, patience, kindness, gentleness of manipulation; close, undivided attention; the mental discipline that gives quickness of perception, and accuracy of comparison and induction; with that easy boldness which quietly assumes nothing to be immodest that is necessary for a full understanding of the nature and extent of the disease, and yet which sacredly avoids all not thus necessary. To place the patient at ease, and at the same time secure attention, it is best to commence the examination with a few leading questions, such as: How long have you been unwell? How did your sickness commence? Do you suffer much pain, and if so, where? Is it sharp, dull, burning, constant or paroxysmal?

Having thus introduced the examination far enough to allow any feeling of trepidation or embarrassment that might have been felt by the patient, to have subsided, and at the same time to have obtained an outline of his particular suffering, you should, without apparent design, pass directly to a methodical examination, so complete as to elicit a correct knowledge of all the important functions and processes performed in the system. By simple inspection you observe the physiognomy or expression; the hue of the skin; the position or attitude; voluntary and involuntary movements; general contour or relative development of parts; and the particular appearance of the tongue, with such other parts as may be the seat of special complaint. All this, except the two last items, may be accomplished while proceeding with the oral part of the examination.

After the leading introductory questions already suggested, the further prosecution of the oral examination should take such direction as to elicit as full an account of the several important functions as the patient is capable of giving. Perhaps the most natural and easy method is to interrogate consecutively concerning the organs engaged in the work of digestion, assimilation, and nutrition; those engaged in the opposite processes of disintegration and excretion; then those constituting the nervous system, both cerebro-spinal and ganglionic; and, finally, those concerned in reproduction, especially in females past the age of puberty. When the patient is too young or too sick to answer properly the necessary inquiries, the same should be directed to the nurse, or whoever has immediate care of the patient. There are some diseases, like those of a typhoid character, that always blunt, more or less, the sensibilities of the patient, and often render the manifestations of mind so inactive as to cause very imperfect or erroneous answers to be given. In other cases we have just the opposite, namely, such an increase of nervous sensitiveness as to cause the most extravagant expressions and the wildest exaggerations. It is proper and desirable always to have the nurse, or some reliable member of the family, present during the examination of such patients, because they will greatly assist in correcting erroneous statements, and in supplying defects in the patient's memory. And it is a good rule in all delicate cases, and such as involve apparent mental derangement, to have a free confidential interview with the nurse alone, during which you can canvass the statements and condition of the patient, without danger of exciting either his suspicions or his anger. I need not say this should be done entirely out of the sight and hearing of the patient. Nothing so quickly excites the fears or the suspicions of a conscious patient as private conversation or *whispering* in his room. All conversation in the presence of the sick should be in a mild, kindly tone of voice, just loud enough to be easily understood, but wholly free from all abrupt and boisterous qualities. The correctness of the information obtained from patients will depend much on the manner in which questions are asked. If they are too general in their character the patient will often fail to comprehend their full meaning, and give erroneous answers in consequence. For instance, many when asked if their food digests well, answer promptly, yes; and yet when asked more particularly, acknowledge that the food often lies heavy in the stomach after eating, or that they have frequent belching of gases, and sometimes acid eructations. So, too, in regard to excretions. I have seen many patients who when asked "if their bowels were regular?" answered without any hesitation, "Yes, they were all right." But when asked specifically how often they had a fœcal evacuation some said once in three or four days; others three or four times every day; while others said once a day. The better way is to ask directly how often the patient has an evacuation from the bowels, and what is the color and consistence of the evacuation. The same rule is still more necessary in obtaining a knowledge of the renal secretion. Unless their attention has been previously called to the subject, many patients will not be able to give a reliable statement either as to the quantity or quality of the urine, but will answer in general terms that they think it is about natural. Others will say they "make a great deal more" than natural, when they really make it very *often*, but only a little at a time. Patients laboring under low forms of fever and paralytic affections, not unfrequently have either partial or complete paralysis of the muscular coat of the bladder. This is liable to cause, first, retention until a certain degree of distention of the bladder, and then dribbling, so as to keep the clothing wet, or the passage of only a few spoonfuls at a time. In all such cases, in addition to careful inquiries of the nurse, the physician should daily examine the hypogastric region sufficiently to determine whether the bladder is distended or not. I have known a neglect of this latter rule to lead to several serious mistakes. It is only a few weeks since that I was requested to see a young man reported to be very dangerously sick from disease of the brain. On calling at the house I met the attending physician, and after listening to a brief history of the case, entered the sick man's room. The patient was entirely comatose; chin dropped; pupils a little dilated; breathing irregular; skin clammy; pulse frequent and very feeble; and frequent irregular muscular twitchings. The latter, with a strong urinous odor about the bed, caused me to inquire whether the patient had passed his water regularly.

The attending physician answered in the affirmative. Turning to the nurse I asked *when* he passed his water last? Her answer was, "He passes it every little while, and his bed is wet now." "How long has he passed his water in the bed?" I inquired, at the same time passing my hand down over the region of the bladder. "Three days," was her reply. The hand at once detected a great degree of fullness in the hypogastrium, which further examination proved to be owing to the presence of a bladder so much distended that its fundus reached the umbilicus. The introduction of a catheter gave exit to an ordinary chambervesselful of ammoniacal urine. The attending physician, not a little chagrined, excused himself by saying he had not examined the region of the bladder because the nurse had assured him every day that the patient had passed his water freely. In most cases of chronic disease presenting obscure questions in relation to their pathology, and especially if accompanied by serous or dropsical effusions, the physician should directly examine the urine, aided both by chemical tests and the microscope.

*Palpation or Touch.*—While the acquisition of an easy, systematic, and accurate method of oral examination is of great importance to the physician, it is never sufficient to give him a full and correct knowledge of the condition of his patient, without the aid of direct contact or touch. By the latter we gain a knowledge of the temperature, and other qualities of the skin; the state of the circulation as indicated by the force, frequency, and regularity of the pulse; the fullness and regularity of respiration; the tension or flaccidity of muscles; the existence of hyperæsthesia and anæsthesia; the existence or non-existence of indurations, enlargements, tumors, abscesses, dropsical effusions, etc.; and the physical condition of the parts within the chest and the abdomen.

In young children, and in patients of all ages whose mental perceptions are disordered by disease, direct physical examination, coupled with inspection, constitute our chief means for acquiring a knowledge of the morbid conditions under which they may be laboring.

*Instrumental Aid.*—To render this part of the examination of patients more complete, various instruments have been constructed, some of which are of great practical value. The ophthalmoscope; otoscope; rhinoscope; laryngoscope; stethoscope; microscope; sphygmograph; thermometer; urinometer; speculums; with test tubes, spirit lamp, and chemical re-agents, constitute the chief instruments which the physician of today may bring to his aid in determining the existence, nature, stage of progress, and tendencies of disease.

I do not say that a physician cannot acquire skill, and even superior skill, both in the diagnosis and treatment of disease, without familiarity with the use of many, and perhaps all of these instruments. And yet it must be admitted that each one of them, properly used, is capable of adding both to the extent and accuracy of our knowledge concerning the morbid conditions it is designed to aid in investigating. It is desirable, therefore, that every general practitioner should be familiar with the use of all these instruments; and as far as practicable, keep them constantly within his reach. No detailed descriptions or illustrative drawings can give you an adequate knowledge of the articles themselves, or of their practical application. Such knowledge can be obtained only by direct examination and actual clinical use.

Happily for you as a class, the daily hospital and dispensary clinics which constitute a prominent part of the course of instruction in this institution, will give you ample opportunities for becoming acquainted, individually, with the practical application of every instrument and appliance that may aid in the examination and treatment of the sick. As already intimated, the primary object of all our examinations of the sick is to ascertain whether they are afflicted by disease, and if so, its nature, extent, duration, etc.—in other words, to arrive at as full and complete a diagnosis as possible.

But what constitutes a complete diagnosis?

Certainly not the mere classification or naming of the disease; for a very superficial examination may enable the practitioner to determine that a patient has typhoid fever, pneumonia, or rheumatism, and yet leave him with a very imperfect knowledge of the pathological changes that had taken place in the solids and fluids of the body. A full and practical diagnosis in any given case embraces, first, a knowledge of the general nature of the disease; second, the pathological changes that have taken place, which determine the stage of advancement; third, the nature and extent of the complications, if any, that have supervened; and, fourth, the physical and mental condition and habits of the patient prior to the present sickness. The first of these items gained will enable you to name the disease; the second and third, to clearly comprehend the present pathological condition of the patient, and found thereon rational indications for treatment; while the fourth will often enable you to anticipate the tendency or direction which other changes will take during the further progress of the case.

The making of a full, practical diagnosis, is, therefore, the most important, and often the most perplexing of all the duties devolving on the medical practitioner. If he succeeds in obtaining a clear and correct knowledge of the nature, progress and tendencies of the disease under which his patient is laboring, it requires but a short and easy process of induction to arrive at the rational indications for treatment: that is, to determine what needs to be done for the purpose of either mitigating or curing the disease, and re-establishing the health of the patient. And, having determined thus logically the indications for treatment which the case requires, a competent knowledge of the principles of hygiene, and of the materia medica, will readily suggest the best means for fulfilling the indications presented. I say a competent knowledge of the principles of *hygiene* as well as of materia medica, because I hope none of you will make so great a mistake as to suppose the treatment of disease consists solely in the administration of drugs.

A large part of the diseases coming under the care of the physician are caused by errors in diet, drinks, clothing, ventilation, and other matters included under the term hygiene; and no one can attain the highest degree of success as a practitioner who does not fully appreciate the importance of careful atttention to the hygienic influences affecting his patients.

The object of such attention is two-fold: namely, to remove or correct such erroneous habits and conditions as may be still acting as causes; and the substitution of such as will positively aid in the restoration of the patient. A comfortable temperature; a sufficient supply of fresh pure air; clean linen; a careful adaptation of the quantity and quality of food and drink to the capacity of the digestive organs to receive and assimilate it; and a quiet, cheerful, hopeful mental influence, are hygienic conditions of universal applicability in the management of the sick. I by no means approve of the modern doctrine of *expectancy*, which assumes that diseases must run their natural course, and that *art* can do little more than properly regulate the hygienic conditions of the patient, and leave the rest to that intangible something called *nature.*

And yet I cannot too strongly urge upon you the importance of making yourselves thoroughly familiar with the facts and principles of public and personal hygiene, and constant attention to their application in the daily routine of practice. It would not be inappropriate to represent hygiene proper as bearing much the same relation to materia medica that physiology does to pathology.

*Therapeutic Methods.*—Before closing this lecture I must invite your attention to a few thoughts concerning the principal therapeutic methods, or systems, as they are sometimes called, that have found advocates among the leading members of the profession in this and the preceding generations. Since the earliest periods of medical history, therapeutics, or the application of remedies in the treatment of disease, have been made to conform more or less closely to the co-existing ideas or doctrines in relation to the nature of disease itself. When the nature and phenomena of diseases were regarded as dependent on certain chemical processes called concoction, fermentation, crices, etc., the prevalent therapeutic system was founded on corresponding chemical notions, and had for its leading objects the hastening of the supposed concoctions, the maturing of the morbid humors, and their expulsion or neutralization.

When the theories of vitalism gained the ascendency, and all diseases were regarded as involving either debility (direct or indirect), or irritation, the prevalent therapeutic ideas were soon found aggregated into two leading and opposing systems. The one, founded on the pathological doctrine of debility, had for its leading object *stimulation*. The other, suggested by the idea of irritation, excitement, etc., had for its purpose diminution of excitement by sedation and evacuation, and hence popularly termed antiphlogistic. During the first quarter of the present century the pathological doctrines of irritation and inflammation gained their most complete control over the mind and practice of the profession. Almost every morbid condition was referred to one or the other of these processes; and, as a consequence, bleeding, general and local, emetics, purgatives, and alteratives, were in constant requisition in the treatment of even the most trivial ailments. But, coincident with this supremacy of the antiphlogistic method in therapeutics, came the rapid development of organic chemistry; the application of the microscope to the study of minute anatomy, both healthy and morbid; and the discovery of the fact that many acute diseases were self-limited in duration, and capable of progressing to recovery without the active interference of art. By the first, our knowledge of the composition and properties of the various morbid products, whether in the tissues, the blood, or the secretions, was greatly increased; and the doctrines of exclusive vitalism began to yield to a recognition of zymotic and blood-diseases. By the second, histological investigations were pushed in every direction, unfolding the minute anatomy of all structures, healthy and morbid, and culminating in the doctrine of *cell* growth as the basis of organic structures, and of the cellular pathology of Virchow. By the third, a distrust or skepticism concerning the curative powers of medicines was rapidly engendered, and a confidence in the restorative processes of nature correspondingly increased.

This tendency soon found marked expression in the writings of Jacob Bigelow, John Forbes, O. W. Holmes, and others; and did not stop until it had effectually checked the heroic use of active remedial agents that had been developed under the preceding doctrines of inflammation and antiphlogistic therapeutics. Under these various influences the former theories or systems of medicine have been abandoned, and yet no other one law, either pathological or therapeutical, has succeeded in gaining any general control over the professional mind. The last twenty years have been characterized by great activity in the accumulation of facts and the multiplication of experiments in almost every department of medical science.

Indeed, it may be regarded as preeminently an era of observation and independent research, untrammeled by authority. And yet, you must not imagine that the present, with all its independence of thought, activity of observation, and vast accumulation of facts, is free from the influence of fanciful theories and bold attempts at generalization. The human mind, in the present, as in all ages of the past, is not only prone to generalize—to frame hypotheses based on a limited number of facts, but, having gained a favorite standpoint, to see all else through light radiating from that focus. Hence, the enthusiastic microscopist, after tracing all organized structures to formation out of primary cells; and structural changes, whether healthy or morbid, to normal and abnormal cell evolutions, naturally enters upon the study of etiology with the favorite instrument in hand, and soon finds organic germs, either animal or vegetable, in the blood, the secretions or the excretions of patients laboring under almost every variety of disease. And these germs are at once heralded as the efficient cause of the diseases with which they are associated. It requires but a hasty glance over the medical literature of the day to see that we have a large class of writers and investigators who are already referring the origin and propagation of cholera, yellow fever, influenza, and other epidemics, as well as many of the endemics, to organic germs. As all these organic germs have their definite periods of development, maturity, propagation and decline, it is consistent and natural to infer that the diseases to which they give rise should also have a definite course to run, which cannot be materially altered by treatment. Hence the *therapeutic* doctrines of this class are fairly expressed in the words palliation and expectancy, while they place great emphasis on hygiene and preventive measures. Another class, with their standpoint of observation in the laboratory of the organic chemist, see in the living system only a complicated series of chemical actions and reactions taking place in the blood, and between the constituents of that fluid and the organized tissues. So long as these processes are well balanced the evolution of caloric, electricity, and nerve force is natural, and health is preserved. But when, from any cause, the equilibrium is disturbed by some change in the chemical factors, the results are also abnormal and disease is established. By this class we have all the ancient doctrines of humoralism revived under the modern terms septicæmia, zymosis, blood degeneration, etc. Their therapeutics are, of course, largely antiseptic and antidotal.

A third class have their standpoint of observation in the physiology and pathology of the nervous tissues, and they find little apparent difficulty in satisfying themselves at least, that almost every variety of action that takes place in living matter, whether healthy or morbid, is under the control of nervous influence.* With such the chief end of therapeutics is to modify the various morbid conditions of structure and function in nerve matter.

But a fourth, and much larger class than either of the foregoing, possessing no definite scientific or theoretical standpoint of observation, being swayed by the general current of reaction from the antiphlogistic system, and captivated, partly by the simplicity of the doctrine that all disease is a diminution of life,† and partly by the plausible eulogiums of "*nature*," and *her* all-controlling power‡ over disease, they have become essentially skeptical in therapeutics, and content to regulate the hygiene of the sick-room, amuse the patient with placebos and wait for "nature" to cure the disease; or, more properly, perhaps, wait for the disease to complete its course and disappear; for we find the greater part of this class not only skeptical in regard to the curative powers of medicines, but also firm believers in the doctrine that diseases have an independent existence, marked by growth, maturity and decline, which makes them, in a great measure, independent of the influence of medication.‖ At a period when investigations are pushed with so much vigor in every direction; when new facts are constantly appearing and old facts are being presented in new aspects; and when so much that is recognized as a part of medical science is but partially or imperfectly known; it is not strange that our literature should be filled with contradictions, better calculated to bewilder than to enlighten the student.

And yet, gentlemen, if you will patiently study the views I have presented to you in the preceding lectures, concerning the elementary forms of disease, the methods of investigation, the indications for treatment, and the principles governing the application of remedies, you will be able to follow me in the study of special pathology and therapeutics in such a way as to become rational and efficient practitioners; neither investing disease with the attributes of independent existence and self-determined duration, nor regarding the curative powers of medicine with a melancholy, vacillating skepticism.

There is one fact in therapeutics that I wish to impress indelibly upon your minds. It is, that the special influence of any and every remedial agent depends much upon the actual condition of the patient at the time it is administered.

For instance: a remedy that, administered in health, or in some conditions of disease, would produce a direct sedative or debilitating influence, if given in some other conditions would result in relieving the sense of oppression and weakness, and add to the strength of the patient. All writers class veratum viride, aconite and digitalis, among the sedatives; yet I have seen many

*See Brown-Sequard's Lecture in the Toner Course, at Washington, D. C., 1873.

†See Chambers.

‡See Essays by Bigelow, Forbes and Holmes.

‖See Dr. Gibson's Address before the British Medical Association, in 1870.

patients so debilitated by insufficient oxygenation and decarbonization of the blood, caused by an irregular, tremulous action of the heart, that they could not walk across the room, who, when placed enough under the influence of these articles to render the heart slow and steady in its beat, could walk or ride with ease. I have seen patients in the first stage of pneumonia, with the face deeply suffused with redness, the breathing short and oppressed, the pulse frequent and weak, and the feeling of prostration so marked that they were unable to rise from the bed, so relieved by one prompt, free bleeding, that they could not only sit up but walk about their room with ease. What are recognized as tonics and stimulants, given to the same patients in the same stage of the disease, instead of strengthening, would have added to the debility of the patients by increasing the local vascular fullness. Again, the same quantity of an anodyne or anæsthetic that might be required simply to render a patient comfortable when suffering from delirium tremens or severe neuralgia, might produce dangerous, or even fatal effects, if given to the same patient when well, or the nervous system not disturbed. Hence, I repeat, that the general effect of any and every remedy will be determined very much by the condition of the patient at the time of its administration.

And I can give you, gentlemen, no more important therapeutical law, or general rule of action, than to so investigate every case as to gain a clear and definite conception of the existing pathological conditions, and then apply such remedies as are most accurately calculated to remove both the morbid conditions and the causes on which they depend, without regard to either nosological arrangements or classifications of the materia medica. When the case is so obscure that a satisfactory idea of the actual morbid conditions cannot be obtained with all the aids for making a proper diagnosis that are within reach, then be content to palliate symptoms as they are presented from day to day, by mild means, rather than hazard doing positive injury by a blind exhibition of more active remedies.

